# COVID 19-Induced Smell and Taste Impairments: Putative Impact on Physiology

**DOI:** 10.3389/fphys.2020.625110

**Published:** 2021-01-26

**Authors:** Nicolas Meunier, Loïc Briand, Agnès Jacquin-Piques, Laurent Brondel, Luc Pénicaud

**Affiliations:** ^1^Université Paris-Saclay, INRAE, UVSQ, VIM, Jouy-en-Josas, France; ^2^Centre des Sciences du Goût et de l’Alimentation, AgroSup Dijon, CNRS UMR6265, INRAE UMR 1324, Université de Bourgogne Franche Comté, Dijon, France; ^3^Department of Clinical Neurophysiology, University Hospital, Dijon, France; ^4^STROMALab, Université de Toulouse, CNRS ERL 5311, Inserm U1031, Université Paul Sabatier (UPS), Toulouse, France

**Keywords:** COVID 19, taste, smell, feeding behavior, physiopathology

## Abstract

Smell and taste impairments are recognized as common symptoms in COVID 19 patients even in an asymptomatic phase. Indeed, depending on the country, in up to 85–90% of cases anosmia and dysgeusia are reported. We will review briefly the main mechanisms involved in the physiology of olfaction and taste focusing on receptors and transduction as well as the main neuroanatomical pathways. Then we will examine the current evidences, even if still fragmented and unsystematic, explaining the disturbances and mode of action of the virus at the level of the nasal and oral cavities. We will focus on its impact on the peripheral and central nervous system. Finally, considering the role of smell and taste in numerous physiological functions, especially in ingestive behavior, we will discuss the consequences on the physiology of the patients as well as management regarding food intake.

## Introduction

In the list of clinical symptoms of COVID-19, a sudden loss of sense of smell and taste has been identified (Mehraeen et al., 2020). This is now recognized as a “significant symptom” that can be found even in the absence of the “usual symptoms” such as fever, cough, respiratory failure. While reports were at first anecdotal and generally without quantitative measurements, a recent study on around 4,000 participants from more than 40 countries confirms that COVID-19 broadly impacts chemosensory function across multiple sensory modalities (Parma et al., 2020). A major reduction in smell, independently of nasal obstruction, and in taste was reported without significant differences between participants tested in laboratory or by clinical assessment via a multi-lingual questionnaire (von Bartheld et al., 2020).

In this review we will describe (i) the main mechanism and neurological pathways underlying olfaction and taste, (ii) the current hypothesis to explain the pathophysiology of anosmia and ageusia, and (iii) the physiological consequences these defects can have, with a focus on feeding behavior.

## Physiology of Olfaction and Taste

Olfaction, taste and chemesthesis are the three separate modalities involved in food flavor perception. Olfaction is involved in the detection of volatile chemical compounds present in the environment or in the oral cavity (by retronasal olfaction), whereas taste (gustation) is involved in the chemical detection of soluble compounds by taste detectors present in taste buds. Chemesthesis, also referred as trigeminal chemosensation, is the chemical sense allowing the detection of another class of taste-related compounds, producing sensations of irritation pungency, burning, tingling or coolness, which can be part of flavor perception (Roper, 2014).

**FIGURE 1:**
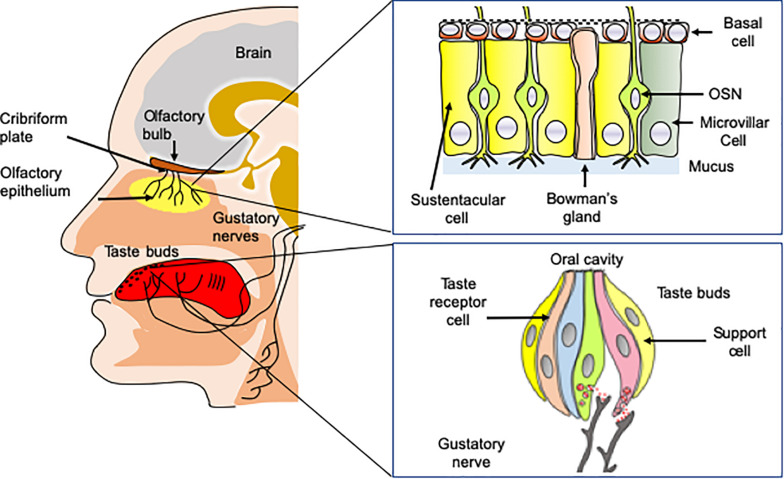
Anatomy of taste and olfaction. Volatile odorant molecules are detected by olfactory sensory neurons (OSNs) located in the olfactory epithelium. OSNs are neurons extending ciliated dendrites in the periphery and with axons forming synapses with glomeruli in the olfactory bulbs situated in the brain. Taste buds located on the tongue are involved in the detection of soluble tasting compounds. The detection is achieved by taste receptor cells located in taste buds. Taste information is transmitted to the brain by three nerves, including chorda tympani, the glossopharyngeal, and the vagus nerves.

Odorant molecules are detected by a complex self-regenerating olfactory epithelium (OE) located in the superior parts of the nasal cavity below the cribriform plate (Figure 1). The OE is composed of several cell types including millions of olfactory sensory neurons (OSNs), in addition to microvillar, sustentacular cells, and basal cells, which are multipotent stem cells (Mombaerts, 2004). OSNs are bipolar neurons extending dendrites over the mucosa surface with axons passing through the cribriform plate to form synapses within glomeruli in the olfactory bulbs. Importantly, the OE is rich in basal stem cells, allowing OSNs to undergo continuous turnover during the life (Kondo et al., 2010). Odorant detection is mediated by a large multigene family that codes for olfactory receptors (ORs). ORs are G protein-coupled receptors (GPCRs) expressed within the membrane of OSN dendrites (Malnic et al., 2004). Myriads of chemically diverse odorants are discriminated in a combinatorial manner in which, one odorant activates a combination of ORs and one OR recognizes multiple odorants (Duchamp-Viret et al., 1999; Malnic et al., 1999). The main components of the canonical signal transduction pathway have been identified. The odorant-bound OR activates the olfactory specific G-protein α subunit, Gα_olf_, which in turn dissociates from Gβγ dimer and activates type III adenylyl cyclase (ACIII). ACIII activation leads to an increased production of cAMP causing the opening of a cyclic nucleotide-gated ion channel (CNG) resulting in neuron depolarization. OSNs project axons to the olfactory bulb located in the brain, where the axons synapse with bulb neurons (mitral and tufted cells). The olfactory information is then transmitted toward a great number of higher brain regions including at first piriform cortex, amygdala, olfactory tubercle, and entorhinal cortex; then to other regions such as orbitofrontal cortex, hypothalamus, thalamus, and hippocampus (Simon et al., 2006; Diodato et al., 2016).

OE is covered by a thin layer of mucus secreted in the olfactory mucosa by the Bowman’s glands (Getchell et al., 1984). The mucus contains large concentrations of odorant-binding proteins (OBPs). OBPs are small soluble proteins secreted in the nasal mucus that reversibly bind odorant molecules (Briand et al., 2002). While their physiological function is not fully understood, they are good candidates for carrying odorants, through the nasal mucus toward the olfactory receptors. The OE contains also many xenobiotic metabolizing enzymes (XMEs). XMEs constitute a large family of enzymes [including Glutathione-S-transferases (GSTs), UDP glucuronosyltransferase (UGT) and cytochrome P_450_ (CYP450)] that are highly expressed in the olfactory epithelium (Heydel et al., 2013). Although their functions in olfaction are still poorly understood, these enzymes are supposed to be involved in odorant transformation, degradation and/or olfactory signal termination (Schwartz et al., 2020).

The sense of taste is essential for the evaluation of the food quality in the oral cavity. It detects nutritive molecules such as carbohydrates or amino acids, electrolytes such as sodium or protons and potentially toxic molecules, which should be avoided (Briand and Salles, 2016). The gustatory system allows perceiving five basic taste qualities, sweet, salty, sour, bitter, and umami (the taste of some amino acids such as L-glutamate and 5’-ribonucleotides). In addition to these five fundamental taste qualities, a number of other taste sensations including fat taste (Laugerette et al., 2005; Mouillot et al., 2019), kokumi (mouthfulness in Japanese) taste (Maruyama et al., 2012) and calcium taste (Behrens et al., 2011; Tordoff et al., 2012) are still a matter of debate. Tasting substances are detected by 2,000–5,000 taste buds, which are located primarily on the tongue, soft palate, and epiglottis in mammals (Briand and Salles, 2016). Taste buds contain specialized taste receptor-cells (TRCs) expressing specific taste receptors, which are stimulated by sapid molecules dissolved in saliva (Behrens et al., 2011). Like OSNs, TRCs are able to undergo continuous renewal throughout the life course (Barlow and Klein, 2015).

The detection of the sweet, bitter, and umami molecules is mediated by G-protein coupled receptors (GPCRs). The sweet taste receptor is composed of two subunits, TAS1R2 (taste receptor type 1, member 2) and TAS1R3 (taste receptor type 1, member 3). These subunits assemble to form a single sweet taste receptor (Nelson et al., 2001) able to detect all the chemically diverse sweet-taste-eliciting chemicals. The bitter tasting compounds are detected in humans by a set of 25 different taste receptors (TAS2Rs) (Meyerhof et al., 2010). Whereas, some bitter receptors respond to only a few bitter compounds, other TAS2Rs are broadly tuned bitter receptors. The umami receptor is a heterodimer composed of TAS1R1 (taste receptor type 1, member 1) and TAS1R3, that assemble to detect the umami tastants (Nelson et al., 2002). The detection of sweet, umami and bitter molecules involves a common transduction mechanism. The main components of this signal cascade have been identified (Iwata et al., 2014). The binding of the tasting compounds to the receptors results in the dissociation of the heterotrimeric G protein (α-gustducin, Gβ3, and Gγ13). The release of the Gβγ protein induces an increase in phospholipase C-β2 (PLC-β2) activity. Activation of PLC-β2 results in the inositol 1,4,5-triphosphate (IP_3_) receptor, type 3-mediated release of calcium from intracellular stores and the gating of a transient receptor potential ion channel, TRPMP5 (Behrens et al., 2018). The epithelial Na^+^ channels (ENaCs) have been proposed to be the sodium receptor (Chandrashekar et al., 2010), whereas, the proton channel Otopetrin-1 has been recently demonstrated to be the sour taste sensor (Tu et al., 2018).

Taste buds are innervated by three nerves, chorda tympani nerve (a branch of the facial nerve CN-VII), the glossopharyngeal (CN-IX) and vagus nerve (CN-X), conveying taste information to the nucleus tractus solitarius (NTS) within the central nervous system. From the NTS, the gustatory information is transmitted to numerous regions including the thalamus, for relay to the primary gustatory cortex located in the somatosensory cortex (Galindo et al., 2012).

The capacity of trigeminal nerve endings located in the nasal and oral cavity to detect the pungent or sharp feel, the coolness, the tingle or the irritation produced by different foods or beverages is called chemesthesis or trigeminal sensitivity (Bryant and Silver, 2000; Viana, 2011). They are detected by transient receptor potential (TRPs) channels, which are present on primary sensory neurons. The information is relayed to the brainstem via trigeminal ganglion sensory neurons (Roper, 2014). Chemesthetic stimuli are transduced by terminals of unmyelinated fibers traveling in trigeminal nerves (V) or by isolated chemosensory cells innervated by afferent axons traveling in these nerves, and possibly by epithelial keratinocytes, as discussed below.

It is then important to review what are the main mechanism by which SARS-COV-2 can affect smell and taste and what are the putative cells infected in these sensory systems.

## SARS-CoV-2 and Anosmia: Cellular Tropism in Olfactory Epithelium

### Cellular Expression of the Virus Receptor

ACE2 (angiotensin-converting enzyme 2) was characterized as the main entrance receptor for SARS-CoV-2 (Letko et al., 2020) interacting with its spike proteins. The spike protein allows the entrance into the host cell via a fusion domain (Delmas and Laude, 1990; Matsuyama et al., 2010). This fusion domain is uncovered after maturation of the spike protein by both ACE2 and the transmembrane serine protease 2 (TMPRSS2). These proteins mainly direct the cellular sensitivity to SARS-CoV-2.

Both proteins are mainly expressed in the upper part of the respiratory tract (Hou et al., 2020) and the highest density of these proteins is found in the olfactory epithelium. Sustentacular cells express most of ACE2 and TMPRSS2 and these proteins are absent from OSN (Bilinska et al., 2020; Fodoulian et al., 2020). Both are also expressed to a lesser extent in Bowman’s gland, microvillar cells and basal stem cells (Brann et al., 2020). Based on this expression profile, sustentacular cells seem to be the main target of the SARS-CoV-2 in the olfactory epithelium.

Interestingly, chemical disorders associated with COVID-19 seem to be linked to the ethnicity. A recent review reporting on nearly 40,000 patients across 104 studies found that anosmia (and ageusia) is more prevalent in Caucasians than Asians (54.8 vs. 17.7%, respectively) (von Bartheld et al., 2020). Such differences in chemical disorder susceptibility do not seem to be due to underreporting, but may be explained by virus strain differences among SARS-CoV-2 (D614G mutation) and/or ethnic variation in the frequencies of ACE2 and/or TMPRSS2 sequences giving more affinity of SARS-CoV-2 to Caucasians (Butowt et al., 2020).

### Cellular Impact: *in vivo* Evidence of SARS-CoV-2 in the Olfactory System

#### Mouse

The first *in vivo* data on the cellular target of the SARS-CoV-2 came from earlier studies of SARS-CoV-1 impact on the central nervous system. These studies are interesting because SARS-CoV-1 and 2 share the same receptor and SARS-CoV-1 has been shown to be neurotropic in studies using ACE2 humanized mice (Netland et al., 2008), thereby raising the possibility that SARS-CoV-2 could infect OSNs. Such infection would open a way for SARS-CoV-2 to enter the brain through the “olfactory pathway” (Bryche et al., 2020a; Forrester et al., 2018) and would explain the prevalence of encephalopathies observed in patients with COVID-19 (Azizi and Azizi, 2020). However, many cells of these humanized mice ectopically express ACE2 as it is under the control of keratin 18 (K18) a promoter of all epithelial cells. SARS-CoV-1 may thus infect OSNs which physiologically do not express ACE2 and the observation of presence of the virus in the brain may not be relevant for a more physiological model. Thus, the mouse—usually favored due to all the different strains and genetic tools available—cannot be directly used to understand the cellular basis of SARS-CoV-2 induced anosmia. Better mouse models are in development to implement a humanized ACE2 with a physiological expression profile (Butowt and von Bartheld, 2020; Sun et al., 2020). Recent studies using this model demonstrates that sustentacular cells and Bowman’s gland cells in the olfactory epithelium are the major targets of SARS-CoV-2 before the invasion into olfactory sensory neurons (Ye et al., 2020; Zheng et al., 2020). Nonetheless other animal models have proved to be relevant to unravel the cellular mechanism behind COVID-19 related anosmia.

#### Golden Syrian Hamsters

Golden Syrian hamsters have been successfully used as a model of SARS-CoV-1 infection (Roberts et al., 2005). Indeed, the expression profile and sequences of ACE2 are very similar in hamsters and humans (Luan et al., 2020). The first study on SARS-CoV-2 impact on hamsters did not focus on anosmia, but provided some information on SARS-CoV-2 presence in the nasal cavity. The authors found that the virus was mainly infecting the olfactory epithelium in the nasal cavity and their results suggested that olfactory sensory neurons may be infected (Sia et al., 2020). Using the same animal model, we published shortly thereafter a study specifically focused on the impact of the SARS-CoV-2 in the nasal cavity (Bryche et al., 2020b). Using confocal double label immunostaining, we observed a massive infection of sustentacular cells by SARS-CoV-2 as early as 2 days post-infection. This infection was accompanied by immune cell infiltration and a global desquamation of the OE. At 2 days post-infection, the lumen of the nasal cavity was filled with cellular aggregates containing infected sustentacular cells, olfactory neurons and immune cells. At 4 days post-infection, the number of infected sustentacular cells was greatly reduced while the olfactory epithelial thickness was reduced up to 80%. Furthermore, the remaining OSNs had mostly lost their cilia involved in odor detection. Seven days post-infection, the virus was almost completely absent from the nasal cavity and we observed a gradual recovery of the olfactory epithelial thickness which reached about 50% of that of the control 14 days after infection. This recovery was also observed for OSN cilia. While we did not measure olfactory based behavior in our study, the massive loss of OSN dendrites undoubtedly had an important impact on odor detection efficiency and could explain most of the observed anosmia symptom if similar cellular events occur in humans. The recovery kinetic is also consistent with the observed recovery of anosmia in COVID-19 patients. Indeed, most patients suffering from anosmia recover relatively fast (∼10 days) (Dell’Era et al., 2020; Meini et al., 2020; von Bartheld et al., 2020), which is compatible with the observed partial recovery of the olfactory epithelium in hamsters 14 days post-infection. These results were later confirmed by another group (Zhang et al., 2020); using a much higher virus load during infection (10^5^ vs. 5.10^3^ pfu in our study). This group looked carefully for a potential infection of OSN. They found that some mature and immature OSN can be infected by SARS-CoV-2 but the study presents only a few images and this infection may be exceptionally rare compared to the occurrence of sustentacular cells infection. The infection of immature neurons could impair regeneration. Similar to other studies (Bryche et al., 2020b; Sia et al., 2020), Zhang et al. (2020) did not find any presence of the virus in the olfactory bulb indicating that if infection of OSN did occur, it did not lead to a detectable presence of the virus in the brain. Thus, so far, the possibility that SARS-CoV-2 could enter the brain through the “olfactory pathway” remains to be demonstrated.

#### Other Animal Models and Human Biopsies

Ferrets are also classically used as a model for respiratory viruses, especially influenza (Belser et al., 2020). The first study on ferrets infected with SARS-CoV-2 did not specifically focus on the olfactory epithelium; however, when they observed the presence of the virus in the nasal cavity, they found only infection of respiratory epithelial cells (Ryan et al., 2020). This result was confirmed in a broader study including fruit bats, pigs and chickens (Schlottau et al., 2020). While both pigs and chicken were resistant to SARS-CoV-2 infection, the fruit bat was susceptible but only few respiratory cells were infected by the virus. The authors observed, however, cellular debris in the lumen of the nasal cavity for both fruit bats and ferrets similarly to reports with infected golden Syrian hamsters.

Data from human biopsies are scarce, and they do not provide a link between the cellular tropism of SARS-CoV-2 to the observed anosmia. Some studies explored olfactory epithelium obtained from autopsied patients with COVID-19 patients. Results are rather controversial so far. A study performed on four samples did not find the virus in the olfactory epithelium by immunohistochemistry (Kantonen et al., 2020). A study based on 33 samples from autopsied patients explored specifically the presence of the virus by RT-qPCR (Meinhardt et al., 2020). They observed the presence of the virus in the olfactory epithelium in 20 patients (∼60%) and in the olfactory bulb in 3 (∼10%). While the authors conclude that SARS-CoV-2 must thus infect OSN allowing it to enter the brain through olfactory bulb invasion, the study does not present any evidence of the presence of infected neurons by immunohistochemistry in the olfactory bulb. Another study focused on biopsies from olfactory epithelium of living COVID-19 positive patients (Chung et al., 2020). In this work, the authors only observed the presence of a few SARS-CoV-2 infected macrophages in the olfactory epithelium but no other cells were found SARS-CoV-2 positive. However, the delay between biopsies and SARS-CoV-2 infection detection was not presented in this study. As biopsies were harvested from patients already suffering from anosmia, it could be that they were performed several days after the onset of the COVID-19 infection and the virus could then be already mostly eliminated from the nasal cavity if a similar kinetic of virus clearance from the nasal cavity occurs in hamsters and humans. If so, earlier human biopsies could be very informative as the virus would be present and impact the olfactory epithelium mostly during the first 4 days following infection. Thus, so far more studies are required to evaluate to which extent the impact of SARS-CoV-2 on the olfactory epithelium differs from the model based on the hamster study. The fact that very few studies observed the presence of SARS-CoV-2 in human OSNs indicates that it may be a rare occurrence (Ellul et al., 2020; Matschke et al., 2020). In any case, it must be noted that the very rapid recovery of smell usually described in both humans and rodents may not be consistent with the timing of olfactory neuron regeneration (which is thought to take 10 or more days; Kondo et al., 2010; Liberia et al., 2019). However, as the onset of infection is very difficult to assess in humans, further studies are required to understand these events.

## Models to Explain COVID-19 Related Anosmia

Overall, most data indicate that the main targets of SARS-CoV-2 in the olfactory epithelium are sustentacular cells. Following their infection, most of the olfactory epithelium seems to be lost by desquamation as indicated by the presence of cellular debris in the lumen of the nasal cavity from numerous studies. This desquamation will remove part of the OSN population but could be accompanied by a loss of the dendrite layer of OSN where olfactory transduction occurs. These two consequences of the SARS-CoV-2 infection could explain the anosmia observed in COVID-19 patient. Subsequently two different scenarios could occur according to the physiological state of the infected individuals as well as the initial virus load. However, in healthy individuals the recovery would be fast due to the basal cells regenerating the olfactory epithelium. This recovery may be impaired by several factors:

• *Individuals characteristics*. Indeed, aged and/or overweight individuals are much more susceptible to COVID-19 (Simonnet et al., 2020). The olfactory epithelium integrity declines with age (Doty and Kamath, 2014) and overweight individual often present an increased basal inflammation state in their tissue (Ellulu et al., 2017) which could also impair regeneration (Chen et al., 2019; Sultan et al., 2011). Infection by SARS-CoV-2 of olfactory epithelium already in an inflammation state may facilitate the virus infection efficiency as its receptor ACE2 is overexpressed during inflammation (Ziegler et al., 2020).
• *Initial virus load.* OSN seems to be infected only with higher virus loads. If this infection reaches a certain threshold, it could begin to affect immature OSNs which will impact the regeneration of the olfactory epithelium.
• *Invasion of the respiratory epithelium*. Part of the olfactory epithelium can be replaced by respiratory epithelium as usually observed in post viral olfactory disorders (Doty and Kamath, 2014). It would diminish the recovery from anosmia.

**FIGURE 2:**
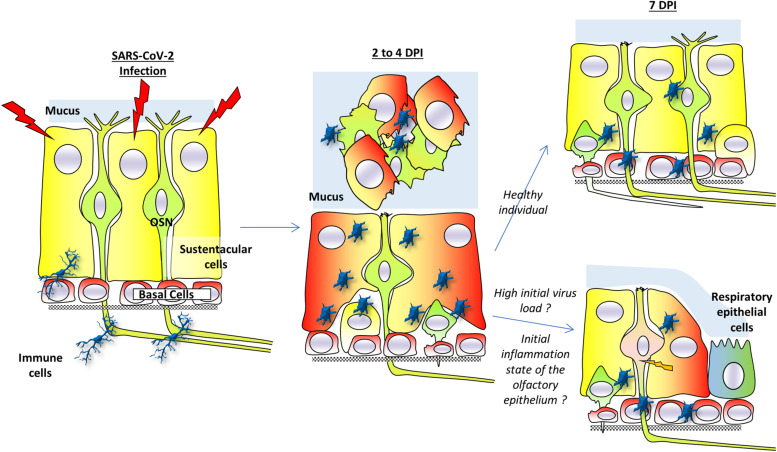
A model for anosmia based on hamsters studies. Sustentacular cells are the main target of SARS-CoV-2 infection. Two to four days post-infection (dpi), the virus is present mainly in these cells. The massive infection of sustentacular cells is followed by a desquamation of the olfactory epithelium which is simultaneously invaded by activated immunity cells. The desquamation is visible through a reduced thickness of the olfactory epithelium and presence of cellular debris in the lumen of the nasal cavity. The cellular aggregates contain sustentacular cells, activated immune cells as well as olfactory neurons. In the non-desquamated zone, the olfactory neurons are losing their dendrite. Seven days post-infection, the olfactory epithelium is already regenerating due to basal cells in healthy individuals and injured olfactory sensory neurons could recover their dendrite layer. If initial virus load is high or in overweight/aged individuals whose initial integrity of the olfactory epithelium may already be impaired, the regeneration of the olfactory epithelium may be less efficient. This could lead to secondary infection of olfactory neurons as well as a slower recovery of the olfactory epithelium function. Part of the olfactory epithelium could also be replaced by respiratory epithelium following the massive inflammation.

This model is summarized in Figure 2. Many questions remain to elucidate the mechanism behind this desquamation.

Is it simply due to the destruction of sustentacular cells following SARS-CoV-2 infection? Indeed, these cells are essential to maintain the integrity of the olfactory epithelium and are tightly enwrapped around olfactory sensory neurons (Liang, 2020). Their disappearance from the olfactory epithelium will certainly impact the olfactory sensory neurons integrity; at least the dendrite layer, if not the cell body as well.

What is the role of the immune cells infiltrating the olfactory epithelium following infection? Are they actively involved in the desquamation of the olfactory epithelium or do they invade the olfactory epithelium following chemo-attractive signals after sustentacular infection and destruction? Indeed, as expected, inflammatory signals are increased in the olfactory epithelium following SARS-CoV-2 infection (Lee et al., 2020).

## SARS-CoV-2 and Ageusia: Cellular Tropism in Taste Buds

Unlike anosmia, COVID-induced ageusia has drawn much less interest in the scientific community, probably because to date, infection of the taste buds has been mostly overlooked. One study on the rabies virus impact in dogs found that taste buds were infected (Shiwa et al., 2018). The virus may reach the taste buds by retrograde transport from the infected brain. Thus, unlike anosmia which could be linked to a potential invasion of the brain through the olfactory nerve, an infection of taste buds which do not contain neurons may not be threatening for the infected individual.

Nevertheless, understanding how SARS-CoV-2 could impact gustation as frequently as olfaction may reveal unsuspected virus-host interactions. ACE2 was suspected to be expressed mainly outside the taste buds (Cooper et al., 2020). This was confirmed by a comprehensive study of the ACE2 expression profile in mice tongue showing that ACE2 is mainly expressed in epithelial cells outside of taste papillae which contain the taste buds (Wang et al., 2020). According to this study, taste buds are thus very unlikely to be directly impacted by the SARS-CoV-2 which may instead infect cells distant from taste buds.

## Models to Explain COVID-19 Related Ageusia

In order to improve our understanding of the cellular basis of ageusia, studies on the oral cavity impact of SARS-CoV-2 in model animals are required. In their absence, only a hypothetical scenario based on other pathological ageusia can be drawn.

One explanation of the SARS-CoV-2 induced ageusia could be that taste nerves are damaged following central nervous infection by SARS-CoV-2. It seems unlikely as a recent study performed in human indicates that impairments of chemical senses are correlated with low severity in COVID-19 patients excluding encephalitis (Nouchi et al., 2020) and the prevalence of central nervous damage by SARS-CoV-2 remains limited (Matschke et al., 2020).

The taste buds have a fast turnover as they are renewed within approximately 10 days (Beidler and Smallman, 1965). Another explanation could thus be that following infection of epithelial cells in the tongue, inflammatory cytokines could reach the taste buds impairing their renewal. Indeed, Toll-like receptors (TLR) and interferon (IFN) receptors are highly expressed in taste buds and their activation may limit taste cell regeneration (Wang et al., 2007, 2009). Thus, ageusia could be the result of impaired renewal of taste buds following the cytokines storm induced by SARS-CoV-2 in distant cells. The cytokine storm could also make taste buds cells permissive to SARS-CoV-2. Indeed, ACE2 has been shown to be overexpressed in the presence of IFN (Ziegler et al., 2020). Thus, a distant production of IFN from infected keratinocytes could lead to ACE2 expression in taste bud cells which could in turn be infected by SARS-CoV-2. A last explanation could be that taste nerves are damaged following central nervous infection by SARS-CoV-2. However, this seems unlikely, because the prevalence of central nervous system damage by SARS-CoV-2 remains limited (Matschke et al., 2020) while the prevalence of ageusia is high. Furthermore, COVID-19 patients suffering from impairments of chemical senses develop low severity symptoms excluding encephalitis (Nouchi et al., 2020).

## Impact of Anosmia and Ageusia in COVID-19 on Feeding Behavior

### Role of Flavor in Eating Behavior in Physiological Conditions

**FIGURE 3:**
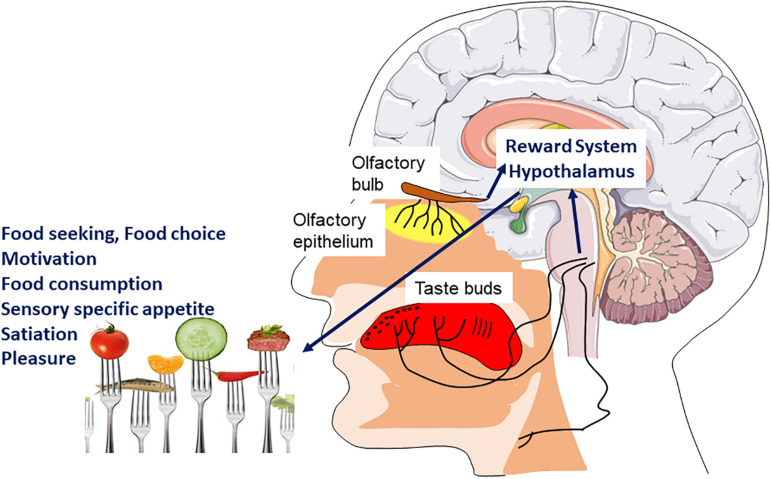
Hedonic sensory signal and food intake. Signals coming from the chemosensory systems (olfaction, taste, and chemesthesis) directly inform the brain of the sensory quality of food. The main brain areas involved are those of the reward system as well as hypothalamic nuclei. These will be involved in different components of feeding behavior such as motivation and pleasure to eat but also sensory specific appetite and satiation.

Smell and taste make an important contribution to the general appetite, food choice, the onset of satiation, thereby participating in the control of energy intake allows an organism to connect the structural and chemical properties of foods to palatability and the foods’ underlying nutritional value (Figure 3). Therefore, the sensory perception resulting from taste, odor and texture of the food, that is, its flavor, allows us to decide to ingest the food or not (Ventura and Worobey, 2013).

Olfaction, despite being perceptually intertwined with taste to produce the food flavor, has different independent physiological mechanisms, neural circuits and effects on food selection and intake (McCrickerd and Forde, 2016). While taste is based on a small class of receptors to detect a few important chemicals of the food once it is in the mouth, olfaction uses many receptors to detect thousands of different smells before and during intake in order to identify a wide variety of foods. Food odor has an important impact on general appetite: it can influence the quality and quantity of food chosen (Fedoroff et al., 1997; Ferriday and Brunstrom, 2008) and stimulate appetite, even in the absence of hunger (Lowe et al., 2009). It has also been suggested that there is a quality-specific effect of each odor that influences food choices (Gaillet-Torrent et al., 2013). Food odors also seem to stimulate sensory specific appetite (Gaillet-Torrent et al., 2014), motivate spontaneous consumption behaviors and help to distinguish different food sources. Some studies have observed that food odors could enhance the onset of satiation and reduce food intake (Ramaekers et al., 2014). Perceived odor intensity during food intake influences the quantity of eaten food (de Wijk et al., 2012): if food odor is perceived more intense, food consumption will be reduced. Food aromas are also signals associated with both food’s availability and pleasure. Consequently, food aromas under fasting conditions and in obesity induce activation of several regions implicated in the reward system according to fMRI’s (functional magnetic resonance imaging) studies (Bragulat et al., 2010; Eiler et al., 2012) and in contrast, decrease their activation in anorexia nervosa (Jiang et al., 2010).

Food taste plays an important role in the control of food intake and taste intensity may stimulate satiation (Bolhuis et al., 2012). In fact, when food enters directly the stomach without being processed by the taste receptors, satiation and reward values are lower (Wijlens et al., 2012; Spetter et al., 2014). Taste is commonly referred to as the body’s “nutritional gatekeeper” of food intake (Feeney et al., 2011). Indeed, the sense of taste is an important factor in food seeking behaviors and dietary intake. Each taste quality has been associated with specific nutrients: sweet taste to identify sources of carbohydrates, sour for the presence of vitamins, salty for the presence of electrolytes and umami for source of proteins (Tucker and Mattes, 2012). In contrast, the bitter taste prevents the ingestion of toxic or spoiled substances (Tucker and Mattes, 2012). All basic tastes, combined with food odors to form flavor, influence food intake and satiation. Numerous studies reported the effect of other taste modalities on the stimulation on food intake. For example, salt enhances palatability and can motivate food intake and then lead to satiation (Bolhuis et al., 2012). Umami is also known to stimulate palatability, appetite, the desire to eat, and therefore food intake (Simpson and Raubenheimer, 2005; Hermanussen et al., 2006). Sweetness contributes to the palatability of food and enhances food intake by increasing its acceptance, especially in children (de Graaf et al., 1993; Mennella and Bobowski, 2015). To illustrate the role of sweet taste, a systematic review reports that in healthy subjects, a strong hedonic preference for sweetness increases the energy intake from sweet foods, especially in subjects with sweet lovers’ phenotypes (Tan and Tucker, 2019). Conversely, a high sensitivity to sweetness (low detection and recognition thresholds) is associated with a low consumption of carbohydrate-rich foods associated with a higher intake of non-sweet foods and dietary protein (Han et al., 2017). Similarly, a strong perception of the intensity of sweetness decreases total energy intake and the consumption of carbohydrate-rich foods (Jayasinghe et al., 2017). These observations indicate that inter-individual differences in sweetness perception (sensitivity and intensity) seem to have a weak and even opposing influence on carbohydrate intakes, in contrast to the sweet induced pleasure which has a great influence on consumption (Tan and Tucker, 2019).

The multisensory properties of food stimuli are transmitted to the brain through specialized taste, olfactory and somatosensory pathways that converge on several central nervous system centers involved in homeostatic and hedonic control of food intake. Hedonic factors that participate in the control of eating behavior by four classic mechanisms (conditioned satiety, food reward system, sensory specific satiety, and alliesthesia) are directly linked to taste and olfaction, and reinforce the flavor pleasantness of food.

### Role of Flavor in Eating Behavior in Pathophysiological Conditions

Taste and smell dysfunctions are common clinical problems associated with disease processes but are often neglected (Henkin et al., 2013). Yet deficits in taste and olfactory chemical senses have a severe impact on the pleasure from foods and represent risk factors for nutritional deficiencies. Causes of smell, taste, and oral somatosensory disorders that affect intakes are numerous: aging, chronic nasal-sinus disease, upper respiratory tract infection, pathologies of the middle ear, head trauma, neurodegenerative disorders, obesity, liver and kidney diseases, cancer, environmental chronic exposures, medications, oral health, surgical interventions, infections and nutritional intervention for chemosensory disorders (for reviews see Schiffman, 1997, 2018; Brondel et al., 2016; Duffy, 2020).

Several studies observed that the coronavirus causing COVID-19 is responsible for smell and taste dysfunctions (Lechien et al., 2020; Parma et al., 2020; von Bartheld et al., 2020). Some studies have reported that 11% of COVID-19 patients with smell loss have chronic deficits, with a chemosensory dysfunction that persists beyond 4 weeks after onset (Boscolo-Rizzo et al., 2020). Accordingly, the current number of such patients worldwide can be estimated. This unprecedent magnitude of the number of cases emphasizes the importance of understanding the clinical consequences of loss of smell/taste.

It was suggested that SARS-CoV-2 is a neurotropic and neuro-invasive virus, by infecting peripheral neurons and then by spreading into the central nervous system like other neuroinvasive viruses (Koyuncu et al., 2013). Concerning the smell functions, the virus may invade the olfactory nerves and the olfactory bulb, causing, on the one hand, olfactory epithelium desquamation and olfactory bulb atrophy, and on the other hand olfactory bulb inflammation (Cooper et al., 2020). Concerning the taste functions, the viral infection and inflammatory response may lead to disruption of saliva composition, taste transduction and impair the continuous renewal of taste buds. Some investigators proposed that the coronavirus causing COVID-19 could also target cells of the central nervous system (Baig et al., 2020). It has also been observed that another coronavirus (SARS-CoV) might enter the central nervous system through the olfactory bulb to spread to some brain areas which are particularly vulnerable to this virus family: piriform and infralimbic cortices, ventral pallidum and lateral preoptic regions in the basal ganglia, and dorsal raphe nuclei in the midbrain (Netland et al., 2008). But as already mentioned a limitation of this study is that it was performed on humanized mice expressing ACE-2 in every epithelial cell.

The influence of hypogeusia (dysgeusia) and/or hyposmia (dysosmia) during COVID-19 on food/energy intake or food preferences has not yet been reported. At the most, literature discusses changes in feeding behavior during the lockdown period, without direct relationship with COVID-19, but in the context of sudden lifestyle changes (Di Renzo et al., 2020; Rodriguez-Perez et al., 2020).

To demonstrate the putative impact of smell and taste disorders in COVID-19 on feeding behavior, we can consider examples of other well-known pathological situations causing the same sensory perturbances. For example, taste and smell alterations resulting from cancers and chemotherapy can reduce appetite and contribute to poor nutritional status (Brisbois et al., 2006; Cohen et al., 2016). In the same way, olfactory dysfunctions in Parkinson disease can lead to changes in feeding behavior (Landis et al., 2009).

Taking into account the potential neurological damage caused by the COVID-19 infection, it is understandable that this virus could have a strong impact on feeding behavior, mediated by taste and smell dysfunctions, and possibly the spreading of the virus to brain regions implicated in hedonic controls of food intake. We can hypothesize that this viral infection, depending on the severity of symptoms, could alter alimentary consumption and nutritional status as in the above-cited pathologies. Indeed, decreases of taste and/or smell may alter the hedonic response associated with the sensory sensations and, therefore, the response to the sensory experience of eating (McCrickerd and Forde, 2016). Putative mechanisms could be a decrease in conditioned satiety (misperception of the aliment before intake), a decrease in the reward system (i.e., low liking and wanting for foods during ingestion) and an early sensory specific satiety (premature termination of the consumed food) (Pénicaud et al., 2016).

The consequence of a decrease in energy intake would then be weight loss (associated with other nutritional imbalances). Studies conducted on animals indicated that SARS-CoV-2 causes weight loss associated with an increase in inflammatory cytokines (Bao et al., 2020). In humans, COVID-19 causes anorexia, weight loss and low albumin levels. The variation between infected individuals is immense; some subjects are asymptomatic or with minimal symptoms, while others develop a severe or even fatal course of the disease. Many factors have been identified in weight loss and sarcopenia/cachexia (Morley et al., 2020). Furthermore, many confounding factors (independent of the increase in energy expenditure related to inflammatory phenomena) may interfere with weight changes linked to taste and smell dysfunctions and food intake reduction, and may be related to change in food and physical activity, sleeping habits, anxiety and depression (Almandoz et al., 2020; Fernandez-Rio et al., 2020; Gualtieri et al., 2020; Ramachandran and Gill, 2020; Zachary et al., 2020). Thus, in absence of studies investigating the direct effect of smell and taste dysfunctions on food intake and preference, it is difficult to quantify precisely their effect in humans. Indeed all these considerations are primarily relevant for the fraction of COVID cases with chronic, not acute loss of smell and taste.

## Conclusion

The relationships between the chemical senses and physiological regulation of food intake are well-known and documented. Even in the absence of relevant studies on the effect of taste and smell alterations on food consumption during COVID-19, special attention should be paid during this period to high-risk individuals with food sensory disturbances, i.e., those with co-morbidities (cardiac, hepatic, and renal), sarcopenia, diabetes, hypertension, smoking, eating disorders, and malnutrition, as well as the elderly. It is crucial to prevent a decrease in food intake during COVID-19 pandemic (Fernandez-Aranda et al., 2020; Pallanti, 2020).

## Author Contributions

NM, LoB, AJ-P, LaB, and LP discussed the concepts, wrote parts, and reviewed the entire manuscript. All authors contributed to the article and approved the submitted version.

## Conflict of Interest

The authors declare that the research was conducted in the absence of any commercial or financial relationships that could be construed as a potential conflict of interest.

## References

[B1] Almandoz J. P. Xie L. Schellinger J. N. Mathew M. S. Gazda C. Ofori A. (2020). Impact of COVID-19 stay-at-home orders on weight-related behaviours among patients with obesity. *Clin. Obes.* 10:e12386. 10.1111/cob.12386 PMC7300461 32515555

[B2] Azizi S. A. Azizi S. A. (2020). Neurological injuries in COVID-19 patients: direct viral invasion or a bystander injury after infection of epithelial/endothelial cells. *J. Neurovirol.* 26 631–641. 10.1007/s13365-020-00903-7 32876900 PMC7465881

[B3] Baig A. M. Khaleeq A. Ali U. Syeda H. (2020). Evidence of the COVID-19 virus targeting the CNS: tissue distribution, host-virus interaction, and proposed neurotropic mechanisms. *ACS Chem. Neurosci.* 11 995–998. 10.1021/acschemneuro.0c00122 32167747

[B4] Bao L. Deng W. Huang B. Gao H. Liu J. Ren L. (2020). The pathogenicity of SARS-CoV-2 in hACE2 transgenic mice. *Nature* 583 830–833. 32380511 10.1038/s41586-020-2312-y

[B5] Barlow L. A. Klein O. D. (2015). Developing, and regenerating a sense of taste. *Curr. Top. Dev. Biol.* 111 401–419. 10.1016/bs.ctdb.2014.11.012 25662267 PMC4435577

[B6] Behrens M. Briand L. de March C. A. Matsunami H. Yamashita A. Meyerhof W. (2018). Structure-function relationships of olfactory and taste receptors. *Chem. Senses* 43 81–87. 10.1093/chemse/bjx083 29342245 PMC6276892

[B7] Behrens M. Meyerhof W. Hellfritsch C. Hofmann T. (2011). Sweet and umami taste: natural products, their chemosensory targets, and beyond. *Angew Chem. Int.* 50 2220–2242. 10.1002/anie.201002094 21337478

[B8] Beidler L. M. Smallman R. L. (1965). Renewal of cells within taste buds. *J. Cell Biol.* 27 263–272. 10.1083/jcb.27.2.263 5884625 PMC2106718

[B9] Belser J. A. Eckert A. M. Huynh T. Gary J. M. Ritter J. M. Tumpey T. M. (2020). A guide for the use of the ferret model for influenza virus infection. *Am. J. Pathol.* 190 11–24. 10.1016/j.ajpath.2019.09.017 31654637 PMC8264465

[B10] Bilinska K. Jakubowska P. Von Bartheld C. S. Butowt R. (2020). Expression of the SARS-CoV-2 entry proteins, ACE2 and TMPRSS2, in cells of the olfactory epithelium: identification of cell types and trends with age. *ACS Chem. Neurosci.* 11 1555–1562. 10.1021/acschemneuro.0c00210 32379417 PMC7241737

[B11] Bolhuis D. P. Lakemond C. M. de Wijk R. A. Luning P. A. de Graaf C. (2012). Effect of salt intensity in soup on ad libitum intake and on subsequent food choice. *Appetite* 58 48–55. 10.1016/j.appet.2011.09.001 21986190

[B12] Boscolo-Rizzo P. Borsetto D. Fabbris C. Spinato G. Frezza D. Menegaldo A. (2020). Evaluation of altered sense of smell or taste in patients with mild symptomatic COVID-19. *JAMA Otolaryngol Head Neck Surg.* 146 729–732. 10.1001/jamaoto.2020.1379 32614442 PMC7333173

[B13] Bragulat V. Dzemidzic M. Bruno C. Cox C. A. Talavage T. Considine R. V. (2010). Food-related odor probes of brain reward circuits during hunger: a pilot fMRI study. *Obesity* 18 1566–1571. 10.1038/oby.2010.57 20339365

[B14] Brann D. H. Tsukahara T. Weinreb C. Lipovsek M. van den Berge K. Gong B. (2020). Non-neuronal expression of SARS-COV-2 entry genes in the olfactory system suggests mechanisms underlying COVID-19 associated anosmia. *Sci. Adv.* 6:eabc5801. 10.1126/sciadv.abc5801 32937591 PMC10715684

[B15] Briand L. Eloit C. Nespoulous C. Bézirard V. Huet J. C. Henry C. (2002). Evidence of an odorant-binding protein in the human olfactory mucus: location, structural characterization, and odorant-binding properties. *Biochemistry* 41 7241–7252. 10.1021/bi015916c 12044155

[B16] Briand L. Salles C. (2016). “Taste perception and integration,” in *Flavor: From Food to Behaviors*, eds Etiévant P. Guichard E. Salles C. Voilley A. (Cambridge: Elservier Ltd.), 101–119. 10.1016/b978-0-08-100295-7.00004-9

[B17] Brisbois T. D. Hutton J. L. Baracos V. E. Wismer W. V. (2006). Taste and smell abnormalities as an independent cause of failure of food intake in patients with advanced cancer–an argument for the application of sensory science. *J. Palliat. Care* 2 111–114. 10.1177/082585970602200208 17265664

[B18] Brondel L. Brindisi M. Jacquin-Piques A. Mouillot T. Pénicaud L. (2016). “Taste disorders in disease,” in *Flavor: From Food to Behaviors*, eds Etiévant P. Guichard E. Salles C. Voilley A. (Cambridge: Elservier Ltd.), 337–361.

[B19] Bryant B. P. Silver W. L. (2000). “Chemesthesis: the common chemical sense,” in *The Neurobiology of Taste and Smell*, eds Finger T. E. Silver W. L. (New York, NY: Wiley-Liss), 73–100.

[B20] Bryche B. Fretaud M. Saint-Albin Deliot A. Galloux M. Sedano L. Langevin C. (2020a). Respiratory syncytial virus tropism for olfactory sensory neurons in mice. *J. Neurochem.* 155 137–153. 10.1111/jnc.14936 31811775

[B21] Bryche B. St Albin A. Murri S. Lacôte S. Pulido C. Ar Gouilh M. (2020b). Massive transient damage of the olfactory epithelium associated with infection of sustentacular cells by SARS-CoV-2 in golden Syrian hamsters. *Brain Behav. Immun.* 89 579–586. 10.1016/j.bbi.2020.06.032 32629042 PMC7332942

[B22] Butowt R. Bilinska K. Von Bartheld C. S. (2020). Chemosensory dysfunction in COVID-19: integration of genetic and epidemiological data points to D614G spike protein variant as a contributing factor. *ACS Chem. Neurosci.* 11 3180–3184. 10.1021/acschemneuro.0c00596 32997488 PMC7581292

[B23] Butowt R. von Bartheld C. S. (2020). Anosmia in COVID-19: underlying mechanisms and assessment of an olfactory route to brain infection. *Neuroscientist* 10.1177/1073858420956905 Online ahead of print. 32914699 PMC7488171

[B24] Chandrashekar J. Kuhn C. Oka Y. Yarmolinsky D. A. Hummler E. Ryba N. J. (2010). The cells and peripheral representation of sodium taste in mice. *Nature* 464 297–301. 10.1038/nature08783 20107438 PMC2849629

[B25] Chen M. Reed R. R. Lane A. P. (2019). Chronic inflammation directs an olfactory stem cell functional switch from neuroregeneration to immune defense. *Cell Stem Cell.* 25 501–513. 10.1016/j.stem.2019.08.011 31523027 PMC6778045

[B26] Chung T. W. Sridhar S. Zhang A. J. Chan K. H. Li H. L. Wong F. K. (2020). Olfactory dysfunction in coronavirus disease 2019 patients: observational cohort study and systematic review. *Open Forum Infect. Dis.* 7:ofaa199. 10.1093/ofid/ofaa199 PMC7284010 32548209

[B27] Cohen J. Wakefield C. E. Laing D. G. (2016). Smell and taste disorders resulting from cancer and chemotherapy. *Cur. Pharmaceutical Design.* 22 2253–2263. 10.2174/1381612822666160216150812 26881441

[B28] Cooper K. W. Brann D. H. Farruggia M. C. Bhutani S. Pellegrino R. Tsukahara T. (2020). COVID-19 and the chemical senses: supporting players take center stage. *Neuron* 107 219–233. 10.1016/j.neuron.2020.06.032 32640192 PMC7328585

[B29] de Graaf C. Schreurs A. Blauw Y. H. (1993). Short-term effects of different amounts of sweet and nonsweet carbohydrates on satiety and energy intake. *Physiol. Behav.* 54 833–843. 10.1016/0031-9384(93)90290-v 8248371

[B30] de Wijk R. Polet I. Boek W. Coenraad S. Bult J. (2012). Food aroma affects bite size. *Flavour* 1:3 10.1186/2044-7248-1-3

[B31] Dell’Era V. Farri F. Garzaro G. Gatto M. Aluffi Valletti P. Garzaro M. (2020). Smell and taste disorders during COVID-19 outbreak: cross-sectional study on 355 patients. *Head Neck* 42 1591–1596. 10.1002/hed.26288 32524707 PMC7300750

[B32] Delmas B. Laude H. (1990). Assembly of coronavirus spike protein into trimers and its role in epitope expression. *J. Virol.* 64 5367–5375. 10.1128/jvi.64.11.5367-5375.1990 2170676 PMC248586

[B33] Di Renzo L. Gualtieri P. Pivari F. Soldati L. Attina A. Cinelli G. (2020). Eating habits and lifestyle changes during COVID-19 lockdown: an Italian survey. *J. Trans. Med.* 18:229. 10.1186/s12967-020-02399-5 PMC7278251 32513197

[B34] Diodato A. Ruinart, de Brimont M. Yim Y. S. Derian N. Perrin S. (2016). Molecular signatures of neural connectivity in the olfactory cortex. *Nat. Commun.* 7:12238. 10.1038/ncomms12238 PMC4960301 27426965

[B35] Doty R. L. Kamath V. (2014). The influences of age on olfaction: a review. *Front. Psychol.* 5:20. 10.3389/fpsyg.2014.00020 24570664 PMC3916729

[B36] Duchamp-Viret P. Chaput M. A. Duchamp A. (1999). Odor response properties of rat olfactory receptor neurons. *Science* 284 2171–2174. 10.1126/science.284.5423.2171 10381881

[B37] Duffy V. (2020). “Causes of smell, taste, and oral somatosensory disorders affecting eating and drinking,” in *Handbook of Eating and Drinking*, ed. Meiselman H. (Berlin: Springer).

[B38] Eiler W. J. Dzemidzic M. Case K. R. Considine R. V. Kareken D. A. (2012). Correlation between ventromedial prefrontal cortex activation to food aromas and cue-driven eating: an fMRI study. *Chemosensory Percept.* 5 27–36. 10.1007/s12078-011-9112-6 25485031 PMC4255712

[B39] Ellul M. A. Benjamin L. Singh B. Lant S. Michael B. D. Easton A. (2020). Neurological associations of COVID-19. *Lancet Neurol.* 19 767–783. 32622375 10.1016/S1474-4422(20)30221-0 PMC7332267

[B40] Ellulu M. S. Patimah I. Khaza’ai H. Rahmat A. Abed Y. (2017). Obesity and inflammation: the linking mechanism and the complications. *Arch. Med. Sci.* 13 851–863. 10.5114/aoms.2016.58928 28721154 PMC5507106

[B41] Fedoroff I. C. Polivy J. Herman C. P. (1997). The effect of pre-exposure to food cues on the eating behavior of restrained and unrestrained eaters. *Appetite* 28 33–47. 10.1006/appe.1996.0057 9134093

[B42] Feeney E. O’Brien S. Scannell A. Markey A. Gibney E. R. (2011). Genetic variation in taste perception: does it have a role in healthy eating? *Proc. Nutr. Soc.* 70 135–143. 10.1017/s0029665110003976 21092367

[B43] Fernandez-Aranda F. Munguia L. Mestre-Bach G. Steward T. Extandi M. Baenas I. (2020). COVID isolation eating scale (CIES) : analysis of the impact of confinement in eating disorders and obesity-A collaborative international study. *Eur. Eat. Disord. Rev*. 28 871–883. 10.1002/erv.2784 32954595 PMC7537123

[B44] Fernandez-Rio J. Cecchini J. A. Mendez-Gimenez A. Carriedo A. (2020). Weight changes during the COVID-19 home confinement. effects on psychosocial variables. *Obes. Res. Clin. Pract.* 14 383–385. 10.1016/j.orcp.2020.07.006 32763110

[B45] Ferriday D. Brunstrom J. M. (2008). How does food-cue exposure lead to larger meal sizes? *British J. Nutr.* 100 1325–1332. 10.1017/s0007114508978296 18466651

[B46] Fodoulian L. Tuberosa J. Rossier D. Boillat M. Kan C. Pauli V. (2020). SARS-CoV-2 receptor and entry genes are expressed by sustentacular cells in the human olfactory neuroepithelium. *iScience* 23:101839. 10.1016/j.isci.2020.101839 33251489 PMC7685946

[B47] Forrester J. V. McMenamin P. G. Dando S. J. (2018). CNS infection and immune privilege. *Nat. Rev. Neurosci.* 19 655–671. 10.1038/s41583-018-0070-8 30310148

[B48] Gaillet-Torrent M. Sulmont-Rossé C. Issanchou S. Chabanet C. Chambaron S. (2013). Priming effects of an olfactory food cue on subsequent food-related behaviour. *Food Qual. Pref.* 30 274–281. 10.1016/j.foodqual.2013.06.008

[B49] Gaillet-Torrent M. Sulmont-Rossé C. Issanchou S. Chabanet C. Chambaron S. (2014). Impact of a non-attentively perceived odour on subsequent food choices. *Appetite* 76 17–22. 10.1016/j.appet.2014.01.009 24462492

[B50] Galindo M. M. Schneider N. Y. Stähler F. Töle J. Meyerhof W. (2012). Taste preferences. *Prog. Mol. Biol. Transl. Sci.* 108 383–426. 22656385 10.1016/B978-0-12-398397-8.00015-0

[B51] Getchell T. V. Margolis F. L. Getchell M. L. (1984). Perireceptor and receptor events in vertebrate olfaction. *Prog. Neurobiol.* 23 317–345. 10.1016/0301-0082(84)90008-x 6398455

[B52] Gualtieri P. Falcone C. Romano L. Macheda S. Correale P. Arciello P. (2020). Body composition findings by computed tomography in SARS-CoV-2 patients: increased risk of muscle wasting in obesity. *Int. J. Mol. Sci.* 21:4670. 10.3390/ijms21134670 32630032 PMC7370146

[B53] Han P. Keast R. S. J. Roura E. (2017). Salivary leptin and TAS1R2/TAS1R3 polymorphisms are related to sweet taste sensitivity and carbohydrate intake from a buffet meal in healthy young adults. *Br. J. Nutr.* 118 763–770. 10.1017/s0007114517002872 29110749

[B54] Henkin R. I. Levy L. M. Fordyce A. (2013). Taste and smell function in chronic disease: a review of clinical and biochemical evaluations of taste and smell dysfunction in over 5000 patients at the taste and smell clinic in Washington. DC. *Am. J. Otolaryngol.* 34 477–489. 23731850 10.1016/j.amjoto.2013.04.006

[B55] Hermanussen M. Garcia A. P. Sunder M. Voigt M. Salazar V. Tresguerres J. A. (2006). Obesity, voracity, and short stature: the impact of glutamate on the regulation of appetite. *Europ. J. Clin. Nutr.* 60 25–31. 10.1038/sj.ejcn.1602263 16132059

[B56] Heydel J. M. Coelho A. Thiebaud N. Legendre A. Le Bon A. M. Faure P. (2013). Odorant-binding proteins and xenobiotic metabolizing enzymes: implications in olfactory perireceptor events. *Anat Rec (Hoboken)* 296 1333–1345. 10.1002/ar.22735 23907783

[B57] Hou Y. J. Okuda K. Edwards C. E. Martinez D. R. Asakura T. Dinnon K. H. III (2020). SARS-CoV-2 reverse genetics reveals a variable infection gradient in the respiratory tract. *Cell* 182 429–446. 32526206 10.1016/j.cell.2020.05.042 PMC7250779

[B58] Iwata S. Yoshida R. Ninomiya Y. (2014). Taste transductions in taste receptor cells: basic tastes and moreover. *Curr. Pharm. Des.* 20 2684–2692. 10.2174/13816128113199990575 23886388

[B59] Jayasinghe S. N. Kruger R. Walsh D. C. I. Cao G. Rivers S. Richter M. (2017). Is sweet taste perception associated with sweet food liking and intake? *Nutrients* 9:750. 10.3390/nu9070750 28708085 PMC5537864

[B60] Jiang T. Soussignan R. Rigaud D. Schaal B. (2010). Pleasure for visual and olfactory stimuli evoking energy-dense foods is decreased in anorexia nervosa. *Psychiatry Res.* 18 42–47. 10.1016/j.psychres.2010.04.041 20488559

[B61] Kantonen J. Mahzabin S. Mäyränpää M. I. Tynninen O. Paetau A. Andersson N. (2020). Neuropathologic features of four autopsied COVID-19 patients. *Brain Pathol.* 30 1012–1016. 10.1111/bpa.12889 32762083 PMC7436498

[B62] Kondo K. Suzukawa K. Sakamoto T. Watanabe K. Kanaya K. Ushio M. (2010). Age-related changes in cell dynamics of the postnatal mouse olfactory neuroepithelium: cell proliferation, neuronal differentiation, and cell death. *J. Comp. Neurol.* 518 1962–1975. 10.1002/cne.22316 20394053

[B63] Koyuncu O. O. Hogue I. B. Enquist L. W. (2013). Virus infections in the nervous system. *Cell Host Microbe* 13 379–393. 23601101 10.1016/j.chom.2013.03.010 PMC3647473

[B64] Landis B. N. Cao Van H. Guinand N. Horvath J. Haehner A. Savva E. (2009). Retronasal olfactory function in Parkinson’s disease. *Laryngoscope* 119 2280–2283. 19753620 10.1002/lary.20547

[B65] Laugerette F. Passilly-Degrace P. Patris B. Niot I. Febbraio M. Montmayeur J. P. (2005). CD36 involvement in orosensory detection of dietary lipids, spontaneous fat preference, and digestive secretions. *J. Clin. Invest.* 115 3177–3184. 10.1172/jci25299 16276419 PMC1265871

[B66] Lechien J. R. Chiesa-Estomba C. M. De Siati D. R. Horoi M. Le Bon S. D. Rodriguez A. (2020). Olfactory and gustatory dysfunctions as a clinical presentation of mild-to-moderate forms of the coronavirus disease (COVID-19): a multicenter European study. *Eur. Arch. Oto-rhino-laryngol.* 277 2251–2261. 10.1007/s00405-020-05965-1 PMC7134551 32253535

[B67] Lee A. C. Zhang A. J. Chan J. F. Li C. Fan Z. Liu F. (2020). Oral SARS-CoV-2 inoculation establishes subclinical respiratory infection with virus shedding in golden syrian hamsters. *Cell Rep. Med.* 1:100121. 10.1016/j.xcrm.2020.100121 32984855 PMC7508015

[B68] Letko M. Marz A. Munster V. (2020). Functional assessment of cell entry and receptor usage for SARS-CoV-2 and other lineage B betacoronaviruses. *Nat. Microbiol.* 5 562–569. 10.1038/s41564-020-0688-y 32094589 PMC7095430

[B69] Liang F. (2020). Sustentacular cell enwrapment of olfactory receptor neuronal dendrites: an update. *Genes (Basel)* 11:493. 10.3390/genes11050493 32365880 PMC7291085

[B70] Liberia T. Martin-Lopez E. Meller S. J. Greer C. A. (2019). Sequential maturation of olfactory sesory neurons in the mature olfactory epithelium. *eNeuro* 6:ENEURO.0266-19.2019. 10.1523/ENEURO.0266-19.2019 31554664 PMC6795559

[B71] Lowe M. R. Butryn M. L. Didie E. R. Annunziato R. A. Thomas J. G. Crerand C. E. (2009). The power of food scale. a new measure of the psychological influence of the food environment. *Appetite* 53 114–118. 10.1016/j.appet.2009.05.016 19500623

[B72] Luan J. Lu Y. Jin X. Zhang L. (2020). Spike protein recognition of mammalian ACE2 predicts the host range and an optimized ACE2 for SARS-CoV-2 infection. *Biochem. Biophys. Res. Commun.* 526 165–169. 10.1016/j.bbrc.2020.03.047 32201080 PMC7102515

[B73] Malnic B. Godfrey P. A. Buck L. B. (2004). The human olfactory receptor gene family. *Proc. Natl. Acad. Sci. U S A.* 101 2584–2589. 14983052 10.1073/pnas.0307882100 PMC356993

[B74] Malnic B. Hirono J. Sato T. Buck L. B. (1999). Combinatorial receptor codes for odors. *Cell* 96 713–723. 10.1016/s0092-8674(00)80581-4 10089886

[B75] Maruyama Y. Yasuda R. Kuroda M. Eto Y. (2012). Kokumi substances, enhancers of basic tastes, induce responses in calcium-sensing receptor expressing taste cells. *PLoS One* 7:e34489. 10.1371/journal.pone.0034489 22511946 PMC3325276

[B76] Matschke J. Lütgehetmann M. Hagel C. Sperhake J. P. Schröder A. S. Edler C. (2020). Neuropathology of patients with COVID-19 in Germany: a post-mortem case series. *Lancet Neurol.* 19 919–929. 10.1016/s1474-4422(20)30308-2 33031735 PMC7535629

[B77] Matsuyama S. Nagata N. Shirato K. Kawase M. Takeda M. Taguchi F. (2010). Efficient activation of the severe acute respiratory syndrome coronavirus spike protein by the transmembrane protease TMPRSS2. *J. Virol.* 84 12658–12664. 10.1128/jvi.01542-10 20926566 PMC3004351

[B78] McCrickerd K. Forde C. G. (2016). Sensory influences on food intake control: moving beyond palatability. *Obesity Rev.* 17 18–29. 10.1111/obr.12340 26662879

[B79] Mehraeen E. Behnezhad F. Salehi M. A. Noori T. Harandi H. SeyedAlinaghi S. (2020). Olfactory and gustatory dysfunctions due to coronavirus disease (COVID-19): a review of current evidence. *Eur. Arch. Otorhinolaryngol.* 17 1–6. 10.1007/s00405-020-06120-6 PMC7297932 32556781

[B80] Meinhardt J. Radke J. Dittmayer C. Mothes R. Franz J. Laueet M. (2020). Olfactory transmucosal SARS-COV-2 invasion as port of central nervous system entry in COVID-19 patients. *Nat. Neurosci.* 10.1038/s41593-020-00758-755 Online ahead of print. 33257876

[B81] Meini S. Suardi L. R. Busoni M. Roberts A. T. Fortini A. (2020). Olfactory and gustatory dysfunctions in 100 patients hospitalized for COVID-19: sex differences and recovery time in real-life. *Eur. Arch. Otorhinolaryngol.* 4 1–5. 10.1007/s00405-020-06102-8 PMC7271634 32500326

[B82] Mennella J. A. Bobowski N. K. (2015). The sweetness and bitterness of childhood: Insights from basic research on taste preferences. *Physiol. Behav.* 152 502–507. 10.1016/j.physbeh.2015.05.015 26002822 PMC4654709

[B83] Meyerhof W. Batram C. Kuhn C. Brockhoff A. Chudoba E. Bufe B. (2010). The molecular receptive ranges of human TAS2R bitter taste receptors. *Chem. Senses* 35 157–170. 10.1093/chemse/bjp092 20022913

[B84] Mombaerts P. (2004). Genes and ligands for odorant, vomeronasal and taste receptors. *Nat. Rev. Neurosci.* 5 263–278. 10.1038/nrn1365 15034552

[B85] Morley J. E. Kalantar-Zadeh K. Anker S. D. (2020). COVID-19: a major cause of cachexia and sarcopenia? *J. Cachexia Sarcopenia Muscle* 1 863–865. 10.1002/jcsm.12589 32519505 PMC7300782

[B86] Mouillot T. Szleper E. Vagne G. Barthet S. Litime D. Brindisi M. C. (2019). Cerebral gustatory activation in response to free fatty acids using gustatory evoked potentials in humans. *J. Lipid. Res.* 60 661–670. 10.1194/jlr.m086587 30587521 PMC6399509

[B87] Nelson G. Chandrashekar J. Hoon M. A. Feng L. Zhao G. Ryba N. J. (2002). An amino-acid taste receptor. *Nature* 416 199–202. 11894099 10.1038/nature726

[B88] Nelson G. Hoon M. A. Chandrashekar J. Zhang Y. Ryba N. J. Zuker C. S. (2001). Mammalian sweet taste receptors. *Cell* 106 381–390. 10.1016/s0092-8674(01)00451-2 11509186

[B89] Netland J. Meyerholz D. K. Moore S. Cassell M. Perlman S. (2008). Severe acute respiratory syndrome coronavirus infection causes neuronal death in the absence of encephalitis in mice transgenic for human ACE2. *J. Virol.* 82 7264–7275. 10.1128/jvi.00737-08 18495771 PMC2493326

[B90] Nouchi A. Chastang J. Miyara M. Lejeune J. Soares A. Ibanez G. (2020). Prevalence of hyposmia and hypogeusia in 390 COVID-19 hospitalized patients and outpatients: a cross-sectional study. *Eur. J. Clin. Microbiol. Infect. Dis.* 8 1–7. 10.1007/s10096-020-04056-7 PMC7543958 33033955

[B91] Pallanti S. (2020). Importance of SARs-COV-2 anosmia: from phenomology to neurobiology. *Compr. Psychiatry* 100:152184. 10.1016/j.comppsych.2020.152184 32422426 PMC7211704

[B92] Parma V. Ohla K. Veldhuizen M. G. Niv M. Y. Kelly C. E. Bakke A. J. (2020). More than smell - COVID-19 is associated with severe impairment of smell, taste, and chemesthesis. *Chem. Senses* 9 609–622. 10.1093/chemse/bjaa041 PMC7337664 32564071

[B93] Pénicaud L. Valentin D. Brondel L. (2016). “Mechanisms involved in the control of feeding behaviour in relation to food flavor”, in *Flavor: From Food to Behaviors*, eds Etiévant P. Guichard E. Salles C. Voilley A. (Cambridge: Elsevier Ltd), 101–119.

[B94] Ramachandran D. Gill T. (2020). Impact of COVID-19 lockdown on self-managed weight loss journeys. *Obes. Res. Clin. Pract.* 14 386–387. 10.1016/j.orcp.2020.08.001 32819875 PMC7413151

[B95] Ramaekers M. G. Luning P. A. Ruijschop R. M. Lakemond C. M. Bult J. H. Gort G. (2014). Aroma exposure time and aroma concentration in relation to satiation. *Br. J. Nutr.* 111 554–562. 10.1017/s0007114513002729 23981570

[B96] Roberts A. Vogel L. Guarner J. Hayes N. Murphy B. Zaki S. (2005). Severe acute respiratory syndrome coronavirus infection of golden Syrian hamsters. *J. Virol.* 79 503–511. 10.1128/jvi.79.1.503-511.2005 15596843 PMC538722

[B97] Rodriguez-Perez C. Molina-Montes E. Verardo V. Artacho R. Garcia-Villanova B. Guerra-Hernandez E. J. (2020). Changes in dietary behaviours during the COVID-19 outbreak confinement in the Spanish COVIDiet study. *Nutrients* 12:1730. 10.3390/nu12061730 32531892 PMC7353108

[B98] Roper S. D. (2014). TRPs in taste and chemesthesis. *Handb. Exp. Pharmacol.* 223 827–871. 10.1007/978-3-319-05161-1_5 24961971 PMC4667542

[B99] Ryan K. A. Bewley K. R. Fotherinngham S. S. Brown P. Hall Y. Marriott A. C. (2020). Dsoe-dependent response to infection with SARS-CoV-2 in the ferret model: evidence of protection ot re-challenge. *bioRxiv* [preprint] 10.1101/2020.05.29.123810 PMC7782478 33398055

[B100] Schiffman S. S. (1997). Taste and smell losses in normal aging and disease. *JAMA* 278 1357–1362. 10.1001/jama.1997.03550160077042 9343468

[B101] Schiffman S. S. (2018). Influence of medications on taste and smell. *World J. Otorhinolaryngol. Head Neck Surg.* 4 84–91. 10.1016/j.wjorl.2018.02.005 30035266 PMC6051304

[B102] Schlottau K. Rissmann M. Graaf A. Schön J. Sehl J. Wylezich C. (2020). SARS-CoV-2 in fruit bats, ferrets, pigs, and chickens: an experimental transmission study. *Lancet Microbe* 1 e218–e225. 32838346 10.1016/S2666-5247(20)30089-6 PMC7340389

[B103] Schwartz M. Menetrier F. Heydel J. M. Chavanne E. Faure P. Labrousse M. (2020). Interactions between odorants and glutathione transferases in the human olfactory cleft. *Chem. Senses* 45 645–654. 10.1093/chemse/bjaa055 32822468

[B104] Shiwa N. Kimitsuki K. Manalo D. L. Inoue S. Park C. H. (2018). A pathological study of the tongues of rabid dogs in the Philippines. *Arch. Virol.* 163 1615–1621. 10.1007/s00705-018-3785-y 29500569

[B105] Sia S. F. Yan L. M. Chin A. W. H. Fung K. Choy K. T. Wong A. Y. L. (2020). Pathogenesis and transmission of SARS-CoV-2 in golden hamsters. *Nature* 583 834–838. 10.1038/s41586-020-2342-5 32408338 PMC7394720

[B106] Simon S. A. de Araujo I. E. Gutierrez R. Nicolelis M. A. (2006). The neural mechanisms of gustation: a distributed processing code. *Nat. Rev. Neurosci.* 7 890–901. 10.1038/nrn2006 17053812

[B107] Simonnet A. Chetboun M. Poissy J. Raverdy V. Noulette J. Duhamel A. (2020). LICORN and the Lille COVID-19 and obesity study group. high prevalence of obesity in severe acute respiratory syndrome coronavirus-2 (SARS-COV-2) requiring invasive mechanical ventilation. *Obesity* 28 1195–1199. 10.1002/oby.22831 32271993 PMC7262326

[B108] Simpson S. J. Raubenheimer D. (2005). Obesity: the protein leverage hypothesis. *Obesity Rev.* 6 133–142. 10.1111/j.1467-789x.2005.00178.x 15836464

[B109] Spetter M. S. Mars M. Viergever M. A. de Graaf C. Smeets P. A. (2014). Taste matters - effects of bypassing oral stimulation on hormone and appetite responses. *Physiol. Behav.* 137 9–17. 10.1016/j.physbeh.2014.06.021 25008799

[B110] Sultan B. May L. A. Lane A. P. (2011). The role of TNF-α in inflammatory olfactory loss. *Laryngoscope* 121 2481–2486. 10.1002/lary.22190 21882204 PMC3540407

[B111] Sun S. H. Chen Q. Gu H. J. Yang G. Wang Y. X. Huang X. Y. (2020). A mouse model of SARS-CoV-2 infection and pathogenesis. *Cell Host Microbe* 28 124–133. 32485164 10.1016/j.chom.2020.05.020 PMC7250783

[B112] Tan S. Y. Tucker R. M. (2019). Sweet taste as a predictor of dietary intake: a systematic review. *Nutrients* 11:94. 10.3390/nu11010094 30621253 PMC6356286

[B113] Tordoff M. G. Alarcón L. K. Valmeki S. Jiang P. (2012). T1R3: a human calcium taste receptor. *Sci. Rep.* 2:496. 10.1038/srep00496 22773945 PMC3390595

[B114] Tu Y. H. Cooper A. J. Teng B. Chang R. B. Artiga D. J. Turner H. N. (2018). An evolutionarily conserved gene family encodes proton-selective ion channels. *Science* 359 1047–1050. 10.1126/science.aao3264 29371428 PMC5845439

[B115] Tucker R. M. Mattes R. D. (2012). Are free fatty acids effective taste stimuli in humans? *J. Food Sci.* 77 S148–S151. 22384969 10.1111/j.1750-3841.2011.02518.x

[B116] Ventura A. K. Worobey J. (2013). Early influences on the development of food preferences. *Curr. Biol.* 23 R401–R408. 23660363 10.1016/j.cub.2013.02.037

[B117] Viana F. (2011). Chemosensory properties of the trigeminal system. *ACS Chem. Neurosci.* 2 38–50. 10.1021/cn100102c 22778855 PMC3369707

[B118] von Bartheld C. S. Hagen M. M. Butowt R. (2020). Prevalence of chemosensory dysfunction in COVID-19 patients: a systematic review and meta-analysis reveals significant ethnic differences. *ACS Chem. Neurosci.* 11 2944–2961. 10.1021/acschemneuro.0c00460 32870641 PMC7571048

[B119] Wang H. Zhou M. Brand J. Huang L. (2007). Inflammation activates the interferon signaling pathways in taste bud cells. *J. Neurosci.* 27 10703–10713. 10.1523/jneurosci.3102-07.2007 17913904 PMC2096741

[B120] Wang H. Zhou M. Brand J. Huang L. (2009). Inflammation and taste disorders: mechanisms in taste buds. *Ann. N. Y. Acad. Sci.* 1170 596–603. 10.1111/j.1749-6632.2009.04480.x 19686199 PMC2729510

[B121] Wang Z. Zhou J. Marshall B. Rekaya R. Ye K. Liu H. X. (2020). SARS-CoV-2 Receptor ACE2 is enriched in a subpopulation of mouse tongue epithelial cells in nongustatory papillae but not in taste buds or embryonic oral epithelium. *ACS Pharmacol. Transl. Sci.* 3 749–758. 10.1021/acsptsci.0c00062 32821883 PMC7409941

[B122] Wijlens A. G. Erkner A. Alexander E. Mars M. Smeets P. A. de Graaf C. (2012). Effects of oral and gastric stimulation on appetite and energy intake. *Obesity* 20 2226–2232. 10.1038/oby.2012.131 22592331

[B123] Ye Q. Zhou J. Yang G. Li R. T. He Q. Zhang Y. (2020). SARS-CoV-2 infection causes transient olfactory dysfunction in mice. *BioRxiv* [preprint] 10.1101/2020.11.10.376673

[B124] Zachary Z. Brianna F. Brianna L. Garrett P. Jade W. Alyssa D. (2020). Self-quarantine and weight gain related risk factors during the COVID-19 pandemic. *Obes. Res. Clin. Pract.* 14 210–216. 10.1016/j.orcp.2020.05.004 32460966 PMC7241331

[B125] Zhang A. J. Lee A. C. Chu H. Chan J. F. Fan Z. Li C. (2020). SARS-CoV-2 infects and damages the mature and immature olfactory sensory neurons of hamsters. *Clin. Infect. Dis.* 10.1093/cid/ciaa995 Online ahead of print. 32667973 PMC7454453

[B126] Zheng J. Wong L. R. Li K. Verma A. K. Ortiz M. Wohlford-Lenane C. (2020). COVID-19 treatments and pathogenesis including anosmia in K18-haCE2 mice. *Nature* 10.1038/41586-020-2943-z Online ahead of print. PMC7855185 33166988

[B127] Ziegler C. G. K. Allon S. J. Nyquist S. K. Mbano I. M. Miao V. N. Tzouanas C. N. (2020). SARS-CoV-2 receptor ACE2 is an interferon-stimulated gene in human airway epithelial cells and is detected in specific cell subsets across tissues. *Cell* 181 1016–1035. 10.1016/j.cell.2020.04.035 32413319 PMC7252096

